# Midventricular Obstruction With Diastolic Paradoxic Jet Flow in Transthyretin Cardiac Amyloidosis: A Case Report

**DOI:** 10.7759/cureus.77097

**Published:** 2025-01-07

**Authors:** Emi Nishiki, Sakiko Honda, Michiyo Yamano, Tatsuya Kawasaki

**Affiliations:** 1 Department of Cardiology, Matsushita Memorial Hospital, Moriguchi, JPN

**Keywords:** amyloidosis, diastolic paradoxic jet flow, midventricular obstruction, physical examination, transthyretin

## Abstract

Transthyretin (ATTR) cardiac amyloidosis has attracted clinical attention because of the development of practical identification and effective treatment. Since one of the initial manifestations of this condition is ventricular hypertrophy, the differential diagnosis includes hypertrophic cardiomyopathy (HCM). Midventricular obstruction (MVO) accompanied by paradoxic jet flow (PJF), which is an early diastolic flow from the apex to the base of the left ventricle, has been observed almost exclusively in HCM. Here, we report a case of ATTR cardiac amyloidosis with MVO and PJF. An 81-year-old woman presented with exertional dyspnea. Echocardiography showed biventricular hypertrophy, MVO with a peak flow velocity of 3.1 m/s in the mid-left ventricle, and PJF. A diagnosis of not HCM but wild-type ATTR cardiac amyloidosis was confirmed by cardiac biopsy and genetic analysis. This case highlights the importance of recognizing MVO along with PJF as a possible morphology in patients with ATTR cardiac amyloidosis.

## Introduction

Transthyretin (ATTR) cardiac amyloidosis is an increasingly recognized disease due to the development of practical identification and effective treatment [1,2]. Echocardiography is a cornerstone modality for suspecting this condition, since ventricular hypertrophy is one of the initial manifestations in patients with ATTR cardiac amyloidosis. The differential diagnosis includes hypertrophic cardiomyopathy (HCM), and more research is needed in this field because cardiovascular outcomes vary in different populations [3].

Midventricular obstruction (MVO) is known as a rare form of HCM [4], in which the presence of paradoxic jet flow (PJF) on echocardiography - an early diastolic flow from the left ventricular apex to the base - has been reported to be associated with MVO [5,6] in various settings [7,8]. We report a case of ATTR cardiac amyloidosis with MVO of the left ventricle accompanied by PJF.

## Case presentation

An 81-year-old woman presented with a four-month history of dyspnea on exertion. She had hypertension, diabetes mellitus, and hyperuricemia. Her medications included candesartan 8 mg daily, vildagliptin 100 mg daily, and allopurinol 100 mg daily. She did not smoke, drink regularly, or use illicit drugs. There was no family history of cardiovascular diseases.

**Figure 1:**
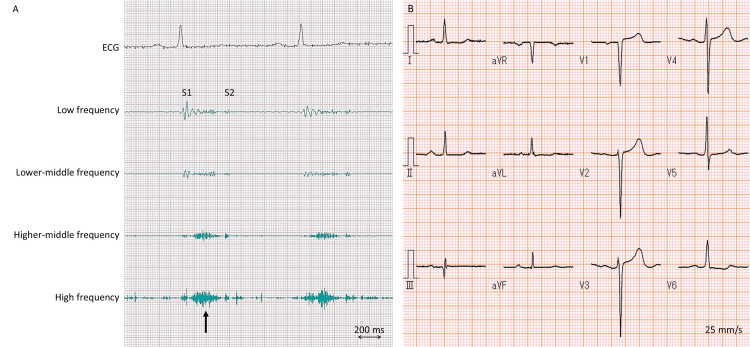
Phonocardiography and electrocardiography. Phonocardiography at the apex shows a high-pitched murmur during early to mid-systole (A, arrow). Note the absence of the fourth sound. Electrocardiography shows no left ventricular hypertrophy (i.e., R-wave amplitude in V5, 1.39 mV) (B). ECG: electrocardiography; S1: the first sound; S2: the second sound

The vital signs were unremarkable. On examination, a grade 2 systolic ejection murmur was heard without additional heart sounds including the fourth sound (Figure 1A). The jugular venous pressure was not elevated. Both lungs were clear on auscultation, and there was no edema in the legs.

Electrocardiography showed slight ST-T changes in leads I, aVL, and V6 without left ventricular hypertrophy (Figure 1B). A chest radiograph was normal. The complete blood count, electrolytes, creatinine kinase level, thyroid function, and renal and liver function tests were normal. The glycated hemoglobin level was 7.1%. The levels of high-sensitivity cardiac troponin T and brain natriuretic peptide were 0.051 ng/mL (reference value, ≤0.100 ng/mL) and 37.7 pg/mL (reference value, ≤18.4 pg/mL), respectively.

**Figure 2:**
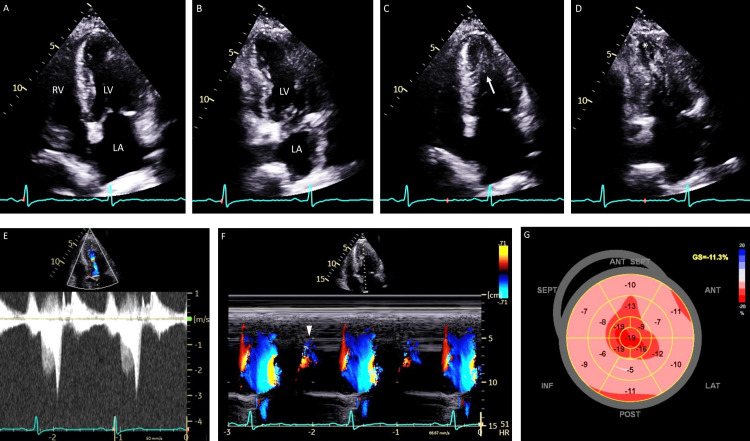
Echocardiography. Apical four- and two-chamber views at end-diastole (A and B) and end-systole (C and D) show mid-ventricular obstruction (C, arrow) with a suspicion of an apical chamber (D, asterisk). Doppler imaging shows a peak velocity of approximately 3.1 m/s in the mid-ventricle (E). Note an early diastolic flow from the apex to the base of the left ventricle during a period of isovolumic relaxation to diastole or paradoxic jet flow (F, arrow). Two-dimensional speckle-tracking echocardiography reveals a global longitudinal strain (GS) of -11.3% with an apical sparing pattern (G). LA: left atrium; LV: left ventricle; RV: right ventricle; HR: heart rate; SEPT: septal; INF: inferior; POST: posterior; LAT: lateral; ANT: anterior; ANT SEPT: anterior-septal

Echocardiography demonstrated a left ventricular ejection fraction of 69% with diffuse hypertrophy in both the left and right ventricles, 17 to 19 mm and 7 to 8 mm, respectively (Figures 2A, 2B). The left ventricular mass index was calculated to be 179.5 g/m^2^ (reference value, 56 to 92 g/m^2^), and the left atrial index was 61 g/m^2^ (reference value, 17 to 32 g/m^2^). Notably, MVO was suspected with a peak velocity of 3.1 m/s (Figures 2C-2E), findings indicative of the presence of an apical chamber, which was confirmed by the presence of PJF (Figure 2F) [5]. Doppler images revealed the ratio of the mitral valve E to A wave of 0.59 and the deceleration time of the E wave of 431 ms, and the ratio of mitral E to early diastolic mitral annular tissue velocity (E/e') was 27.14 on the septal side and 26.51 on the lateral side. Two-dimensional speckle-tracking echocardiography demonstrated a global longitudinal strain of -11.3% with an apical sparing pattern (Figure 2G).

**Figure 3:**
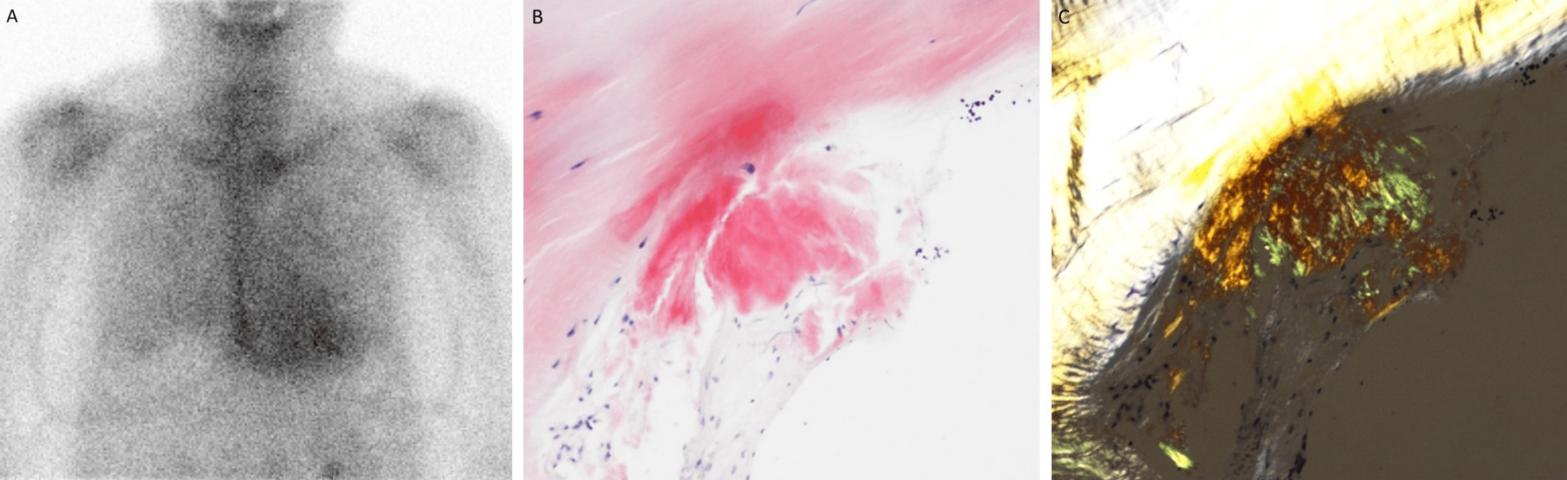
Scintigraphic and pathological findings. Technetium-99m hydroxymethylene diphosphonate scintigraphy shows diffuse uptake in the left ventricle more than in the bone (A). Endomyocardial biopsy of the right ventricle shows positive deposits on Congo red staining (B; original magnification, ×200) and apple green appearance on polarizing microscopy (C; original magnification, ×200).

Further history revealed that the patient had had surgery for flexor tendinitis three years earlier. The patient could not make a neat circle with the thumb and index finger, known as the perfect O sign, findings suggestive of carpal tunnel syndrome. Technetium-99m hydroxymethylene diphosphonate scintigraphy showed diffuse uptake in the left ventricle that was greater than that in the bone (Figure 3A), findings consistent with ATTR cardiac amyloidosis [1]. The biopsy specimen obtained from the intraventricular septum was positive for apple green birefringence on Congo red staining under polarized light (Figures 3B, 3C); the type of the amyloid was confirmed as ATTR on immunohistochemistry performed at the University Hospital Kyoto Prefectural University of Medicine using the anti-TTR115-124 antibody (donated by the Amyloidosis Research Committee, Research Program on Rare and Intractable Diseases, Health, Labour and Welfare Sciences Research Grants, Japan). A diagnosis of wild-type ATTR cardiac amyloidosis was made on the genetic analysis.

Treatment with tafamidis was initiated, and the patient was scheduled for an outpatient visit to the cardiac and orthogeriatric departments.

## Discussion

We had a case of wild-type ATTR cardiac amyloidosis with MVO of the left ventricle. Furthermore, diastolic PJF was confirmed by Doppler echocardiography. To our knowledge, this unique flow has been exclusively reported in patients with HCM.

The classic description of cardiac morphology in cardiac amyloidosis is concentric symmetric hypertrophy, but asymmetric septal hypertrophy, which is traditionally typical of HCM, is unlikely to be a rare pattern of ventricular remodeling in patients with ATTR cardiac amyloidosis [9]. In a study of 263 consecutive patients with ATTR cardiac amyloidosis and 50 with amyloid light-chain (AL) amyloidosis [10], asymmetric septal hypertrophy, defined as a septal to posterior wall ratio >1.5, was present in 79% of patients with ATTR cardiac amyloidosis, whereas patients with AL amyloidosis showed the incidence of 14%. Although the pattern of asymmetric septal hypertrophy should be noted (i.e., 70% sigmoid septum and 30% reverse septal contour; the latter is typical of HCM), the low incidence of symmetric left ventricular hypertrophy (i.e., 18%) in patients with ATTR cardiac amyloidosis should be kept in mind to avoid missing ATTR cardiac amyloidosis on a first-line modality to assess cardiac morphology, such as echocardiography.

In the present patient, the left ventricular morphology was not asymmetric septal hypertrophy but left ventricular MVO, which is known as a rare form of HCM. MVO of the left ventricle, defined as a peak gradient of ≥30 mmHg, has been reported to be found in 46 (9.4%) of 490 patients with HCM [4]. It is worth noting that the diagnosis of MVO may be missed on echocardiography because of inadequate apical images. Missed or delayed diagnosis of this condition should be avoided since left ventricular MVO has been reported to be an independent predictor of adverse outcomes [4]. Cardiac magnetic resonance (MR) is a more sophisticated imaging modality, but its use is limited by lack of availability, cost, long acquisition time, claustrophobia, and contraindications such as metallic implants and non-MR-compatible devices.

The presence of PJF may be useful in the early recognition of MVO, as shown in the current patient. PJF is known to be a sign of apical aneurysm [5] because this unique flow is reported to be caused by an increased pressure gradient between the high-pressure apical chamber and low-pressure basal portion of the left ventricle during isovolumic relaxation and early diastole [5,6]. Clinical data regarding the effect of MVO on ATTR cardiac amyloidosis are lacking, but the current patient should be followed closely given the risk of MVO in HCM. Further research is needed to investigate the clinical implication of the presence of MVO in patients with ATTR cardiac amyloidosis.

The present patient had no fourth heart sound, which was confirmed by both auscultation and phonocardiography. The absence of the fourth sound despite the presence of left ventricular hypertrophy in sinus rhythm may be valuable in suspecting an underlying disease for left ventricular hypertrophy. The incidence of the fourth heart sound is high up to 79% in patients with HCM [11], whereas ATTR amyloidosis had a low incidence of the fourth sound of 47% [12]. The exact mechanism of the difference between the two entities remains unknown, but reduced atrial kick as a result of amyloid infiltration in the atria has been proposed [13]. In addition to the absence of the fourth sound, other physical findings may play an important role in the diagnostic process of ATTR cardiac amyloidosis, such as the inability to make a perfect O sign, as shown in our patient, and Popeye's sign, a bunching of the arm on flexion due to a non-traumatic rupture of the biceps tendon.

## Conclusions

We had a case of wild-type ATTR cardiac amyloidosis with MVO and PJF of the left ventricle. She could not make a neat circle with her thumb and index finger and had no fourth heart sound despite ventricular hypertrophy in sinus rhythm. The present case highlights the importance not only of recognizing MVO with PJF as a possible morphology in patients with ATTR cardiac amyloidosis but also of a focused physical examination in the diagnosis of patients with unexplained left ventricular hypertrophy.
